# Supplementary material for: Expression of the Populus Orthologues of AtYY1, YIN and YANG Activates the Floral Identity Genes AGAMOUS and SEPALLATA3 Accelerating Floral Transition in Arabidopsis thaliana
Source: Int J Mol Sci. 2023 Apr 21;24(8):7639. doi: 10.3390/ijms24087639 (PMC10146089; doi:10.3390/ijms24087639)
Supplement: Supplementary file 1 [file ijms-24-07639-s001.zip › ijms-2243465-supplementary.pdf]

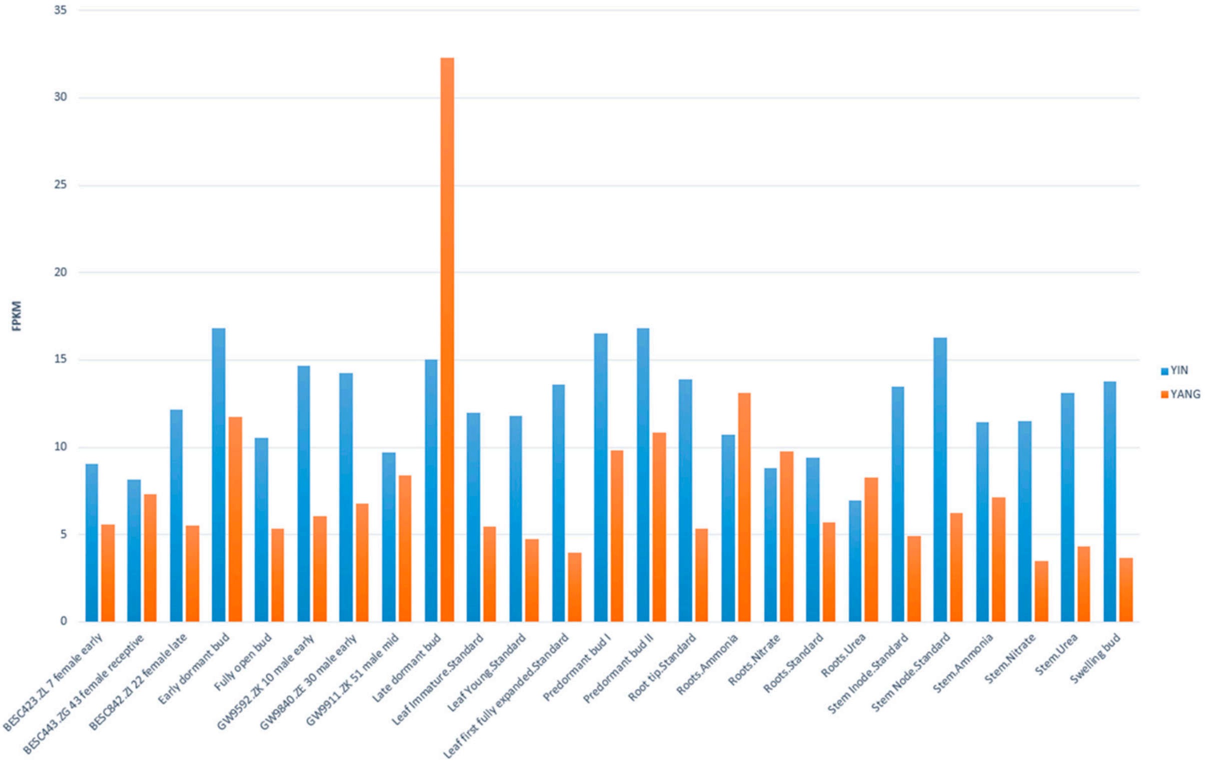

Supplementary Figure S1. Published gene expression data of *YIN* and *YANG* in different tissues of Populus (*Populus trichocarpa*) from the Website phytozome-next.jgi.doe.gov.

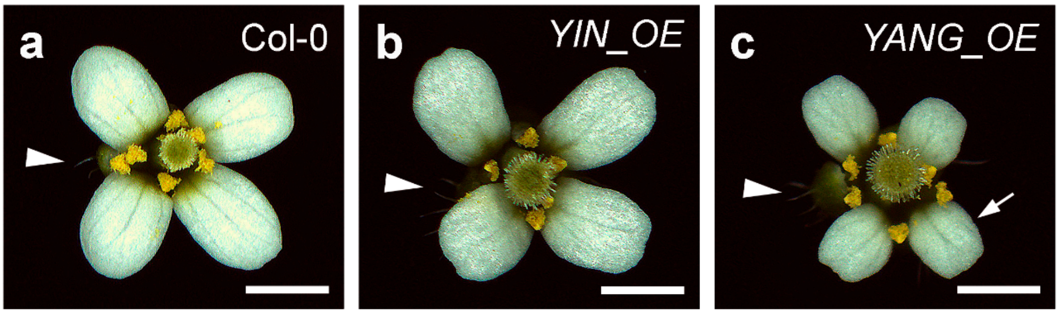

Supplementary Figure S2. Flower phenotype of Arabidopsis *YIN_OE* and *YANG_OE* lines. Arrow heads mark unbranched trichomes that are characteristic for sepals. Arrow: Some but not all flowers in *YIN_OE* and *YANG_OE* lines had smaller Petals. Scale bars = 2 mm.

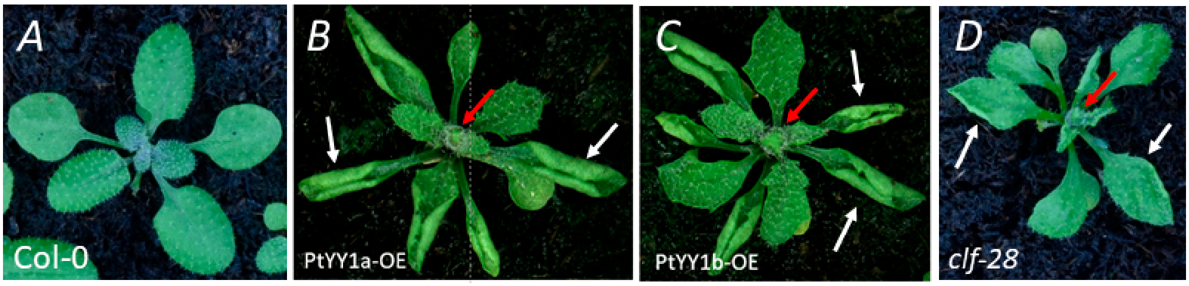

Supplementary Figure S3. In the T1 generation, some *YIN_OE* (PtYY1a-OE, B) and *YANG_OE* (PtYY1b-OE, C) plants displayed earlier flowering and stronger leaf curling than the transgenic plants in the T3 and T4 generation (Figure 5). Note that in the transgenic plants (B-C) rather the juvenile rosette leaves (white arrows) are stronger curled than the later produced cauline leaves at the shoot (red errors), while in loss of *CLF* function mutants (*clf-28*, D), it is the other way around.

Supplementary Table S1. Accession numbers and source species for *YY1* homologs from the Websites phytozome-next.jgi.doe.gov used for the phylogenetic analysis in Figures 1 and 2.

Species & Protein name in Figure 1	Gene ID	Phytozome Genome ID & internet link (https://phytozome-next.jgi.doe.gov/)	Abbreviation in Figure 2	Plant Family
Arabidopsis thaliana YY1	AT4G06634.1	167 (https://phytozome-next.jgi.doe.gov/info/Athaliana_TAIR10)	AtYY1	*Brassicaceae*
Populus trichocarpa YIN (YY1a)	Potri.004G221700.1	444 (https://phytozome-next.jgi.doe.gov/info/Ptrichocarpa_v3_1)	YIN	*Salicacese*
Populus deltoides YY1a	Podel.04G226500.1	445 (https://phytozome-next.jgi.doe.gov/info/PdeltoidesWV94_v2_1)	PdYY1a	*Salicacese*
Salix purpurea YY1a	Sapur.001G179500.1	519 (https://phytozome-next.jgi.doe.gov/info/Spurpurea_v5_1)	SpYY1a	*Salicacese*
Populus trichocarpa YANG (YY1b)	Potri.003G010400.1	444 (https://phytozome-next.jgi.doe.gov/info/Ptrichocarpa_v3_1)	YANG	*Salicacese*
Populus deltoides YY1b	Podel.03G008000.1	445 (https://phytozome-next.jgi.doe.gov/info/PdeltoidesWV94_v2_1)	PdYY1b	*Salicacese*
Salix purpurea YY1b	Sapur.003G005600.2	519 (https://phytozome-next.jgi.doe.gov/info/Spurpurea_v5_1)	SpYY1b	*Salicacese*
Ricinus communis YY1	30093.m000368	444 (https://phytozome-next.jgi.doe.gov/info/Ptrichocarpa_v3_1)	-	*Euphorbiaceae*
Gossypium hirsutum YY1a	Gohir.A01G182000.1	458 (https://phytozome-next.jgi.doe.gov/info/Ghirsutum_v1_1)	-	*Malvaceae*
Gossypium hirsutum YY1b	Gohir.D01G173100.1	458 (https://phytozome-next.jgi.doe.gov/info/Ghirsutum_v1_1)	-	*Malvaceae*
Theobroma cacao YY1	Thecc.02G025800.1	523 (https://phytozome-next.jgi.doe.gov/info/Tcacao_v2_1)	-	*Sterculiaceae*

Corymbia citriodora YY1	Cocit.G0117.1	507 (https://phytozome-next.jgi.doe.gov/info/Ccitriodora_v2_1)	-	*Myrtaceae*
Carya illinoinensis YY1a	CiLak.05G051000.1	561 (https://phytozome-next.jgi.doe.gov/info/CillinoinensisLakota_v1_1)	-	*Juglandaceae*
Carya illinoinensis YY1b	CiLak.06G144300.1	561 (https://phytozome-next.jgi.doe.gov/info/CillinoinensisLakota_v1_1)	-	*Juglandaceae*
Vitis vinifera YY1	VIT_212s0035g00660.2	457 (https://phytozome-next.jgi.doe.gov/info/Vvinifera_v2_1)	-	*Vitaceae*
Carica papaya YY1	evm.model.supercontig_161.1	113 (https://phytozome-next.jgi.doe.gov/info/Cpapaya_ASGPBv0_4)	-	*Caricacese*
Cucumis sativus YY1	Cucsa.076000.1	122 (https://phytozome-next.jgi.doe.gov/info/Csativus_v1_0)	-	*Cucurbitaceae*
Prunus persica YY1	Prupe.1G576100.1	298 (https://phytozome-next.jgi.doe.gov/info/Ppersica_v2_1)	-	*Rosacese*
Fragaria x ananassa YY1a	snap_masked-Fvb5-4-processed-gene-170.22	675 (https://phytozome-next.jgi.doe.gov/info/Fxananassa_v1_0_a1)	-	*Rosacese*
Fragaria x ananassa YY1b	maker-Fvb5-2-augustus-gene-182.29	675 (https://phytozome-next.jgi.doe.gov/info/Fxananassa_v1_0_a1)	-	*Rosacese*
Fragaria x ananassa YY1c	maker-Fvb5-3-snap-gene-92.43	675 (https://phytozome-next.jgi.doe.gov/info/Fxananassa_v1_0_a1)	-	*Rosacese*
Coffea arabica YY1	evm.model.Scaffold_634.202	453 (https://phytozome-next.jgi.doe.gov/info/Carabica_v0_5)	-	*Rubiales*
Phaseolus vulgaris YY1	Phvul.002G248200.1	442 (https://phytozome-next.jgi.doe.gov/info/Pvulgaris_v2_1)	-	*Fabaceae*
Glycine soja YY1a	GlysoPI483463.05G140800.1	509 (https://phytozome-next.jgi.doe.gov/info/Gsoja_v1_1)	-	*Fabaceae*
Glycine soja YY1b	GlysoPI483463.08G116400.1	509 (https://phytozome-next.jgi.doe.gov/info/Gsoja_v1_1)	-	*Fabaceae*

Supplementary Table S2. List of primers used in this study

Gene name	Primer name	Primer sequences (5 => 3)	
PtActin 7	PtACTIN7-qF	TGTTGCCCTTGACTATGAGCAGGA	RT-RT-qPCR
	PtACTIN7-qR	ACGGAATCTCTCAGCTCCAATGGT	RT-RT-qPCR
YIN	YIN-qF	CACCTAATGCTGACAATGAA	RT-RT-qPCR
	YIN-qR	CTGTCTTCTTCTTCTTCATA	RT-RT-qPCR
	YIN_F	AACA**GGTCTC**AGGCTCAATGGAGGCTCATCATCATCAT	cloning
	YIN_R	AACA **GGTCTC** ACTGA GTCTTCATCCTCCGTCTCTTC	cloning
YANG	YANG-qF	ATTCTAATGCTGATAATGAG	RT-RT-qPCR
	YANG-qR	CTTCACTGTCTTCTTCGTAA	RT-RT-qPCR
	YANG_F	AACA**GGTCTC**AGGCTCAATGGAGGCTCATCACCATCAT	cloning
	YANG_R	AACA**GGTCTC**ACTGAGTCTTCATCCTCTGTCTCTTC	cloning
AteIF4A1	AteIF4A1_qF	TTCGCTCTTCTCTTTGCTCTC	RT-RT-qPCR
	AteIF4A1_qR	GAACTCATCTTGTCCCTCAAGTA	RT-RT-qPCR
AtFT	AtFT_qR	CTGTTTGCCTGCCAAGCTGTC	RT-RT-qPCR
	AtFT_qF	CCTCAGGAACTTCTATACTTTGGTTATGG	RT-RT-qPCR
AtAG	AtAG_qF	TCCGATCCAAGAAGAATGAG	RT-RT-qPCR
	AtAG_qR	TGCTCGTAGTTAGATCCTCC	RT-RT-qPCR
AtSEP3	AtSEP3_qF	CTTCAAGAGAGGCCTTAAGCAGTTGA	RT-RT-qPCR
	AtSEP3_qR	TTGTCTCCAGTCAGCATGCGTT	RT-RT-qPCR
AtSOC1	AtSOC1_qF	ACGAGAAGCTCTCTGAAAAGTGGG	RT-RT-qPCR
	AtSOC1_qR	CTTGGGCTACTCTCTTCATCACCT	RT-RT-qPCR
AtLFY	AtLFY_qF	TCTCCCAAGAAGGGTTATCTG	RT-RT-qPCR
	AtLFY_qR	TCTTCATCTTTCCTTGACCTG	RT-RT-qPCR

Note: Bold and underlined sequences in the cloning primers are restriction enzyme sites
